# Research on emergency management of urban waterlogging based on similarity fusion of multi-source heterogeneous data

**DOI:** 10.1371/journal.pone.0270925

**Published:** 2022-07-07

**Authors:** Huimin Xiao, Liu Wang, Chunsheng Cui

**Affiliations:** Henan University of Economics and Law, Zhengzhou, China; Universita degli Studi del Molise, ITALY

## Abstract

Global warming has seriously affected the local climate characteristics of cities, resulting in the frequent occurrence of urban waterlogging with severe economic losses and casualties. Aiming to improve the effectiveness of disaster emergency management, we propose a novel emergency decision model embedding similarity algorithms of heterogeneous multi-attribute based on case-based reasoning. First, this paper establishes a multi-dimensional attribute system of urban waterlogging catastrophes cases based on the Wuli-Shili-Renli theory. Due to the heterogeneity of attributes of waterlogging cases, different algorithms to measure the attribute similarity are designed for crisp symbols, crisp numbers, interval numbers, fuzzy linguistic variables, and hesitant fuzzy linguistic term sets. Then, this paper combines the best-worst method with the maximal deviation method for a more reasonable weight allocation of attributes. Finally, the hybrid similarity between the historical and the target cases is obtained by aggregating attribute similarities via the weighted method. According to the given threshold value, a similar historical case set is built whose emergency measures are used to provide the reference for the target case. Additionally, a case of urban waterlogging emergency is conducted to demonstrate the applicability and effectiveness of the proposed model, which exploits historical experiences and retrieves the optimal scheme for the current disaster emergency with heterogeneous multi attributes. Consequently, the proposed model solves the problem of diverse data types to satisfy the needs of case presentation and retrieval. Compared with the existing model, it can better realize the multi-dimensional expression and fast matching of the cases.

## 1. Introduction

Nowadays, urban waterlogging occurs in various countries frequently due to abnormal climate, destructed eco-environment, and human activities. Modern cities appear extremely vulnerable to heavy rain and floods, which dramatically increases the difficulty of preventing and controlling urban floods because of irresponsible urban planning and poor infrastructure for waterlogging prevention. Urban waterlogging disasters have brought catastrophic damages to infrastructures and people’s lives worldwide. Not only urban waterlogging but various types of emergencies could cause severe consequences. It continually reminds governments of the importance of emergency decision-making. Hence, to improve the efficiency of emergency decision-making, it is crucial to build a suitable emergency decision model suitable for the characteristics of emergencies.

Many researchers have spent considerable effort on emergency decisions and have achieved significant breakthroughs. In public health emergencies, existing research provided the model which combined the best-worst method (BWM) and data envelopment analysis to select the reasonable sites of makeshift hospitals [1] and the method for interactive multi-criteria group decision-making with probabilistic linguistic information to select emergency assistance areas [2]. Geetha et al. proposed a fuzzy case-based reasoning approach for finding COVID-19 patients’ priority in hospitals during the source shortage period [3]. Wan et al. developed a personalized individual semantic-based consensus reaching process and applied it to select COVID-19 surveillance plans [4]. Cui et al. proposed a new group decision-making method to select the right COVID-19 epidemic prevention and control programs [5]. Based on case-based reasoning(CBR), a model embedding the grey relational analysis and the grey wolf optimization algorithms was proposed for engineering emergency decisions [6]. In the disaster emergency, a bi-objective trapezoidal fuzzy mixed-integer linear program model was designed for emergency logistics locations after the earthquake [7]. Yu et al. proposed a method integrating case-based reasoning with intelligent algorithms to support disaster emergency management [8]. An emergency decision-making model for environmental emergencies was constructed based on CBR to deal with gasoline explosion accidents [9].

From the above studies, emergency events are characterized by urgent time and serious consequences. Because of the limitations of experience and knowledge, humans cannot make an optimal decision in the case of limited time. Compared with other methods, CBR is more suitable for on-site optimization of emergency responses, which can provide a referential solution for decision-makers in the shortest time by analyzing prior experience. CBR has been widely applied in many domains, not only including emergency management [6, 8, 9] but also in complex artificial intelligence [10–13], waste treatment [14], and biological domain [15]. In construction projects, CBR helped a lot in risk management and estate valuation [16, 17]. Qin et al. [18] proposed an ontology-supported case-based reasoning approach for computer-aided tolerance specification. Thus, considering the advantages and success of similar applications in various fields, CBR appears to be a promising method for emergency management in urban waterlogging.

In the practical CBR applications, both the historical cases stored in the case base and the target case are described by multiple attributes, and the formats of the attribute values are various. For example, Geetha et al. [3] introduced a fuzzy CBR approach and used fuzzy linguistic variables to represent case attributes. A novel model for predicting traffic emission was proposed using interval-valued intuitionistic fuzzy sets and CBR theory [19]. Zheng et al. [20] developed a new case retrieval method for hybrid multi-attribute, which involved interval numbers and intuitionistic fuzzy numbers. With the diversification of data types, a single data type cannot satisfy the needs of case presentation. Many scholars used heterogeneous data to represent case attributes, including crisp symbols, crisp numbers, and fuzzy numbers [21–23]. The description of urban waterlogging cases is complicated, so it is necessary to consider various forms of data. For example, the warning level of urban waterlogging can be represented by crisp symbols, and crisp numbers describe rainfall. Meanwhile, transportation, electrical, and communication equipment under urban waterlogging need to be represented by hesitant fuzzy linguistic term sets.

Because hesitancy is a very common problem in decision-making, we use hesitant fuzzy linguistic term sets to increase the flexibility of eliciting and representing linguistic information. The theory of fuzzy sets was proposed in 1965 by Zadeh. Then, Torra [24] introduced hesitant fuzzy sets, a generalization of fuzzy sets that permits us to represent the situation in which different membership functions are considered possible. Further, Xu and Xia [25] studied the distance and similarity of hesitant fuzzy sets. Farhadinia [26] proposed the systematic transformation of the entropy into the similarity measure for hesitant fuzzy sets. Verma [27] studied some new properties arising from operations on hesitant fuzzy sets. However, uncertainty is produced by the vagueness of meanings whose nature is qualitative rather than quantitative. For such cases, Rodríguez et al. [28] proposed the concept of the hesitant fuzzy linguistic term set. Then, Liao et al. [29] studied the distance and similarity measures for hesitant fuzzy linguistic term sets.

Usually, the retrieval of the CBR method is achieved based on the similarity measure between the historical cases and the target case. Thus, the study on the similarity measure method in case retrieval is necessary. In the research of similarity measures, Li et al. [30] introduced some similarity measures for the fuzzy environment. A new method was developed by calculating the similarity between paths to retrieve previously performed routes based on CBR [31]. With the uncertainty of features in cases, a hybrid similarity measure for case retrieval was designed to solve the problem of data diversification in the CBR method [20–23].

Although these approaches are effective in dealing with emergency decision-making problems, there are still some research gaps:

1. There is no research on emergency management of urban waterlogging. Due to the changeable decision-making environment and the difficulty in attribute selection, it is urgent to establish an effective model for assessing the urban waterlogging situation.
2. Previous CBR studies generally consider one or two forms of data in case presentation. Now, the description of cases is increasingly complicated, so a single data type cannot satisfy the needs of case presentation. The attributes of the cases should be described by different data types, such as crisp symbols, crisp numbers, interval numbers, fuzzy linguistic variables, and hesitant fuzzy linguistic term sets. Especially, an extensive review of CBR literature reveals few kinds of research on heterogeneous multi-attribute decision-making involving hesitant fuzzy linguistic term sets.
3. There are hybrid attribute values for a case in CBR practical application. Performing case retrieval with high accuracy for multiple formats of attribute values is a significant challenge, but an in-depth study is lacking. It is necessary to design different matching algorithms for diverse data types. Especially, the similarity measure for hesitant fuzzy linguistic term sets in existing studies ignored the hesitance degree, which leads to information loss and distortion.
4. Because of the abundance of different types of attributes, it is difficult to elicit appropriate attribute weights from human experts. Therefore, determining criteria weight vectors in heterogeneous multi-attributes decision-making problems becomes another research gap.

In order to fill these research gaps, this paper aims to research emergency management of urban waterlogging based on similarity fusion of multi-source heterogeneous data. The contributions of our work are as follows. First, we establish a multi-dimensional attribute system of urban waterlogging disaster cases based on WSR theory. Second, in the case presentation process, we use five different data types to describe the situation of urban waterlogging cases, including crisp symbols, crisp numbers, interval numbers, fuzzy linguistic variables, and hesitant fuzzy linguistic term sets. At the same time, different matching algorithms are designed for heterogeneous attributes to calculate local similarity. We propose a new method to calculate the similarity of hesitant fuzzy linguistic term sets. Third, we develop a new method of weighting that combines the BWM with the maximal deviation method, which integrates the subjective and objective factors. Finally, attribute similarities for the five formats are aggregated using the weighted method to form a hybrid similarity. Based on these results, a heterogeneous multi-attribute emergency decision-making model based on CBR is developed to retrieve the optimal scheme for the current disaster emergency.

The paper is organized as follows: Section 2 constructs a case database of urban waterlogging, which introduces a multi-dimensional attribute system, case presentation, and data sources. Section 3 presents the construction of an emergency decision-making model based on CBR and the decision-making process. In section 4, case studies of urban waterlogging emergencies are conducted to validate the method’s effectiveness. Section 5 provides some numerical results and comparisons to justify the algorithm. Finally, conclusions and suggestions for further research are summarized in Section 6.

## 2. Establishment of a case base

### 2.1 Multi-dimensional heterogeneous attributes system of urban waterlogging

This paper adopts a paradigm of WSR system approach studies, which emphasizes the synthesis of perspectives toward problem description and analysis to determine attributes of urban waterlogging cases. “Wuli-Shili-Renli (WSR)” as a system approach is a methodology of integrated system engineering, a typical system thinking of Chinese traditional philosophical views [32]. Wuli refers to the nature and mechanism of things from natural science. Shili is how people can use the mechanism of things to guide the practice through the understanding of Wuli, that is, to provide an answer to the problem. Renli entails the analysis of the influence and relationship between people with various functions in the system, combined with the traditional cultural, legal, and other factors, to study how to give full play to the initiative in practice. It is better to aim to connect WSR to get a comprehensive scenario of what is concerned and find a satisfactory and feasible result. When applying the WSR approach, we emphasize knowing Wuli, sensing Shili, and caring for Renli.

According to the current study on the construction of the urban waterlogging case and the experience of urban waterlogging emergency management, this paper analyzes attributes of urban waterlogging from the perspectives of WSR. The result shows that thirteen factors should be considered to present the case of urban waterlogging. In addition, these attributes are presented by five types of data, as shown in Table 1.

**Table 1:** Attributes system of urban waterlogging.

Analysis perspective	Attributes	Data Type of the Attributes
Wuli	Duration of rainfall(h)	crisp number
	Rainfall total(mm)	crisp number
	Maximum rainfall per hour (mm/h)	crisp number
	Reservoir operation	fuzzy linguistic variable
	State of embankment	fuzzy linguistic variable
	Depth of surface accumulated water	interval number
Renli	Direct economic losses(billion)	crisp number
	Casualties	crisp number
	Range of traffic disruption	hesitant fuzzy linguistic term set
	Range of communication outage	hesitant fuzzy linguistic term set
	Range of power outage	hesitant fuzzy linguistic term set
Shili	Early-warning level	crisp symbol
	Relief supplies demand	hesitant fuzzy linguistic term set

### 2.2 Case presentation and data sources

Case presentation mainly presents the historical enforcement cases and the target enforcement case according to a specific format, which provides the basis for the CBR process. An appropriate case presentation method can improve the efficiency of extracting historical cases and enhance the accuracy of the results of waterlogging case analysis. In this paper, the case presentation is composed of the case attributes and the emergency measures.

Assume that *Z* = {*Z*_1_, *Z*_2_, … *Z*_*n*_} stands for the set of historical cases and *C*^*o*^ represents the target case, in which *Z*_*i*_ represents the number *i* historical case, *i* ∈ *N* = {1, 2, …, *n*}. Let *X* = {*X*_1_, *X*_2_, …, *X*_*m*_} be the collection of the attributes of waterlogging cases, where *X*_*j*_ represents the number *j* attribute in the case, *j* ∈ *M* = {1, 2, …, *m*}. At the same time, *x*_*i*_ = {*x*_*i*1_, *x*_*i*2_, …, *x*_*im*_} and *c* = {*c*_*o*1_, *c*_*o*2_, …, *c*_*om*_}, *j* ∈ *M* represent the set of attributes of historical cases and target case, respectively. Assume that *y*_*i*_ = {*y*_*i*1_, *y*_*i*2_, …, *y*_*il*_} stands for the collection of emergency measures of each historical case, where *y*_*ik*_ represents the number *k* measure in the number *i* case, *k* = 1, 2, …*l*. Let *ω* = (*ω*_1_, *ω*_2_…*ω*_*m*_)^*T*^ be the weight vector of the attributes of the urban waterlogging case, where *ω*_*j*_ is the weight of the *j*^*th*^ attribute of the case. Meanwhile, the attribute values of waterlogging cases can be expressed in the forms of crisp symbols, crisp numbers, interval numbers, fuzzy linguistic variables, and hesitant fuzzy linguistic term sets.

Nowadays, society is one era of information explosion, and how to fully obtain various data from different sources to serve people is becoming more critical. Therefore, the historical case data of this study is from multiple sources, including existing documents, browsing through emergency management-related websites, and using the crawler technology to pull data. Because of the inconsistency of multi-sources data, it is necessary to carry on the operations of choosing data, proving data, and revising data to produce integrated information. In this paper, data pre-processing is done through python programming and Excel.

## 3. Construction of emergency decision-making model based on CBR

### 3.1 Local similarity measurement for heterogeneous attributes

Urban waterlogging attributes are measured by five types of value formats, crisp symbols, crisp numbers, interval numbers, fuzzy linguistic variables, and hesitant fuzzy linguistic term sets, as shown in Table 1. This study extends the applicability of similarity measures of the classic CBR from single-category attributes to heterogeneous multi-attributes. In measuring the similarity between the two cases, local similarities are found by measuring the similarity of each attribute. Then, these local similarities are aggregated to calculate hybrid similarity by using the weights. Therefore, this study constructs different similarity algorithms to calculate the local similarity between attributes regarding five different data types. At the same time, *c*_*oj*_ and *x*_*ij*_ are the attribute values of historical case *Z*_*i*_ and target case *C*^*o*^, respectively.

1. Crisp symbols
  Crisp symbols are enumeration values. When the attribute value is a crisp symbol, the only thing to do is to compare the same factors of the target case and historical cases, so the following calculation formula can be obtained:
$$sim_{cs}(c_{oj},x_{ij})=\left\{\begin{array}{c} 1,c_{oj}=x_{ij}\\ 0,c_{oj}\neq x_{ij} \end{array}\right.,i\in N,j\in M$$ (1)
2. Crisp numbers
  When we use crisp numbers to describe the attributes, the distance-based method can be employed to measure the similarity between the historical and the target cases, and the formula is [33]:
$$\begin{array}{cc} sim_{cn}(c_{oj},x_{ij})=\text{exp}\left[\frac{-|c_{oj}-x_{ij}|}{d_{j}^{\text{max}}-d_{j}^{\text{min}}}\right], & i\in N,j\in M \end{array}$$ (2)
  where,
$$\begin{array}{l} d_{j}^{\text{max}}=\text{max}\{c_{oj},\text{max}\{x_{ij}\}\}\\ d_{j}^{\text{min}}=\text{min}\{c_{oj},\text{min}\{x_{ij}\}\} \end{array}$$
3. Interval numbers
  Interval numbers are used to describe the uncertainty of the attribute value. When attributes are presented by interval numbers, $c_{oj}=[c_{oj}^{l},c_{oj}^{u}]$ and $x_{ij}=[x_{oj}^{l},x_{oj}^{u}]$ are the attribute values of the target case and historical case, respectively. The formula is [34]:
$$sim_{in}(c_{oj},x_{ij})=1-\frac{\int_{x_{ij}^{l}}^{x_{ij}^{u}}\int_{c_{oj}^{l}}^{c_{oj}^{u}}\left|x-y\right|dydx}{\left(c_{oj}^{u}-c_{oj}^{l})(x_{ij}^{u}-x_{ij}^{l})(U-L\right)},i\in N,j\in M$$ (3)
4. Fuzzy linguistic variables
  For the attributes of fuzzy linguistic variables, values are linguistic variables such as very bad(VB), bad(B), medium(M), good(G), and very good(VG). Each of these linguistic variables is represented by a triangular fuzzy number and these numbers are (0, 0, 0.2), (0.1, 0.3, 0.4), (0.3, 0.5, 0.7), (0.6, 0.7, 0.9), (0.8, 1.0, 1.0), respectively. In the following equations [35, 36], $c_{oj}=[c_{oj}^{l},c_{oj}^{m},c_{oj}^{u}]$ and $x_{ij}=[x_{ij}^{l},x_{ij}^{m},x_{ij}^{u}]$ are the attribute values of the target case and historical case:
$$\begin{array}{c} sim_{fl}(c_{oj},x_{ij})=1-\frac{\left|c_{oj}^{l}-x_{ij}^{i}\right|+\left|c_{oj}^{m}-x_{ij}^{m}\right|+\left|c_{oj}^{u}-x_{ij}^{u}\right|}{3}\\ =1-\frac{1}{3}d(c_{oj},x_{ij}) \end{array}$$ (4)
5. Hesitant fuzzy linguistic term sets

**Definition 1** [37]. Let *x*_*i*_ ∈ *X*(*i* = 1, 2, …, *n*), be fixed and *S* = {*s*_*t*_|*t* = −*τ*, …, −1, 0, 1, …*τ*} be a linguistic term set. A hesitant fuzzy linguistic term set (HFLTS) on *X*, is in mathematical terms of *H*_*S*_ = {〈*x*_*i*_, *h*_*S*_(*x*_*i*_)〉|*x*_*i*_ ∈ *X*}, where *h*_*S*_(*x*_*i*_)is a set of some values in the linguistic term set *S* and can be expressed as *h*_*S*_(*x*_*i*_) = {*s*_*ϕn*_(*x*_*i*_)|*s*_*ϕn*_(*x*_*i*_) ∈ *S*, *n* = 1, …, *N*} with *N* being number of linguistic terms in *h*_*S*_(*x*_*i*_). *h*_*S*_(*x*_*i*_) expresses the possible degrees of linguistic variable *x*_*i*_ to the linguistic term set *S*. *h*_*S*_(*x*_*i*_) is called the hesitant fuzzy linguistic element (HFLE) and *H*_*S*_ is the set of all HFLEs.

**Definition 2** [29]. Let *b* = {*b*_*l*_|*l* = 1, …, #*b*} be an HFLTS (#b is the number of linguistic terms in b), *b*^+^ and *b*^−^ be the maximum and minimum linguistic terms in b respectively, and *ς*(0 ≤ *ς* ≤ 1) be an optimized parameter. Then we can add the linguistic term $\bar{b}=\varsigma b^{+}+(1-\varsigma )b^{-}$ into the HFLTS.

**Definition 3**. Let *S* = {*s*_*t*_|*t* = −*τ*, …, −1, 0, 1, …, *τ*} be a linguistic term set, $h_{s}^{1}=\{s_{\delta_{l}^{1}}|l=1,\ldots ,\#h_{s}^{1}\}$ and $h_{s}^{2}=\{s_{\delta_{l}^{2}}|l=1,\ldots ,\#h_{s}^{2}\}$ be two hesitant fuzzy linguistic elements. For many distance measures for HFLTSs, the difference in number of linguistic terms between two HFLEs is ignored. This paper improves the distance measure of HFLTSs based on this. The hesitance degree of the hesitant fuzzy linguistic elements is defined as:

$$d_{h_{s}^{1},h_{s}^{2}}=1-\frac{L(h_{s}^{1}\cap h_{s}^{2})}{L(h_{s}^{1}\cup h_{s}^{2})}$$ (5)

where $L(h_{s}^{1}\cap h_{s}^{2})$ is number of linguistic terms in the intersection of $h_{s}^{1}$ and $h_{s}^{2}$, as well as $L(h_{s}^{1}\cup h_{s}^{2})$ being number of linguistic terms in the union of $h_{s}^{1}$ and $h_{s}^{2}$.

**Definition 4**. For the hesitant fuzzy linguistic elements $h_{s}^{1}$ and $h_{s}^{2}$ in Definition 3, $\#h_{s}^{1}$ and $\#h_{s}^{2}$ are number of linguistic terms in $h_{s}^{1}$ and $h_{s}^{2}$, respectively. If $\#h_{s}^{1}=\;\#h_{s}^{2}$ (otherwise, the shorter one will be extended by adding the linguistic terms through the method in Definition 2.), let the linguistic terms in $h_{s}^{1}$ and $h_{s}^{2}$ be arranged in ascending order. Combining the distance measure for HFLTSs in [29] with the hesitance degree of the hesitant fuzzy linguistic elements in Definition 3, this paper proposes a hybrid similarity measurement, such as improved Hamming hesitant fuzzy linguistic similarity:

$$sim_{h}(h_{s}^{1},h_{s}^{2})=1-0.5\left[d_{h_{s}^{1},h_{s}^{2}}+\frac{1}{L}\sum_{l=1}^{L}\frac{\left|\delta_{l}^{1}-\delta_{l}^{2}\right|}{2\tau +1}\right]$$ (6)

and Euclidean similarity between $h_{s}^{1}$ and $h_{s}^{1}$:

$$sim_{e}(h_{s}^{1},h_{s}^{2})=1-{\left\{0.5\left[d^{2}{}_{h_{s}^{1},h_{s}^{2}}+\frac{1}{L}{\sum_{l=1}^{L}\left(\frac{\left|\delta_{l}^{1}-\delta_{l}^{2}\right|}{2\tau +1}\right)}^{2}\right]\right\}}^{\frac{1}{2}}$$ (7)

Thus, the generalized similarity between $h_{s}^{1}$ and $h_{s}^{1}$ can be defined as:

$$sim(h_{s}^{1},h_{s}^{2})=1-{\left\{0.5\left[d^{\lambda }{}_{h_{s}^{1},h_{s}^{2}}+\frac{1}{L}{\sum_{l=1}^{L}\left(\frac{\left|\delta_{l}^{1}-\delta_{l}^{2}\right|}{2\tau +1}\right)}^{\lambda }\right]\right\}}^{\frac{1}{\lambda }}$$ (8)

Where *λ>0*.

Particularly, if *λ* = 1, the above generalized similarity becomes the Hamming similarity. If *λ* = 2, the above generalized similarity becomes the Euclidean similarity.

### 3.2 Determination of weights

The weights of each attribute are vital to hybrid similarity measurement, which directly affects the result of case reasoning. There are so many methods of weight determination, such as the analytic hierarchy process [16], the Delphi method [32], and the maximal deviation [38].

A new approach integrating subjective and objective weights is proposed in this study. It calculates two weights for each of the criteria. The subjective weight is obtained using the best worst method. At the same time, the objective weight is obtained using the maximal deviation method. Then, the final weight is calculated as a combination of these two weights.

#### 3.2.1 A linear model of BWM

The BWM, a recently developed intuitive and robust multi-criteria decision-making technique, was applied to determine the weights of the decision criteria [39]. The original BWM involves a nonlinear model that sometimes results in multiple optimal weights meaning that the weight of each criterion is presented as an interval. In some cases, a unique solution is preferred. Therefore, this paper adopts a linear model of BWM whose linearity results in a unique solution. The steps followed in BWM to derive the weights of the decision criteria are as follows:

**Step 1**: Determine a set of decision criteria. A set of decision criteria {*X*_1_, *X*_2_, …, *X*_*m*_} are identified, which are used to make a decision.
**Step 2**: Determine the best criteria (B) and the worst criteria (W). The experts choose the B and W criteria arbitrarily.
**Step 3**: Establish the best-to-others (BO) vector. The preference of the best criterion over all the other criteria is determined using a number between 1 and 9. Then, the BO vector would be given: *A*_*B*_ = (*a*_*B*1_, *a*_*B*2_, …, *a*_*Bm*_), and *a*_*Bj*_ shows the preference of the *B* over criterion *j*. Obviously, *a*_*BB*_ = 1.
**Step 4**: Establish the others-to-worst (OW) vector. The preference of all the criteria over the worst criterion is determined using a number between 1 and 9. Then, the OW vector would be given: *A*_*W*_ = (*a*_1*W*_, *a*_2*W*_, …, *a*_*mW*_)^*T*^, where, *a*_*jW*_ shows the preference of the criterion *j* over the worst criterion *W*. Obviously, *a*_*WW*_ = 1.
**Step 5**: In order to determine the optimal weights of the criteria *ω*′ = (*ω*_1_, *ω*_2_…*ω*_*m*_)^*T*^, the following linear programming problem should be solved.
$$\begin{array}{l} \text{min}\;\xi^{L}\\ s.t.\\ \left|\omega_{B}^{\prime }-a_{Bj}\omega_{j}^{\prime }\right|\leq \xi^{L},\text{for}\;\text{all}\;\text{j}\\ \left|\omega_{j}^{\prime }-a_{jW}\omega_{W}^{\prime }\right|\leq \xi^{L},\text{for}\;\text{all}\;\text{j}\\ \sum_{j}\omega_{j}^{\prime }=1\\ \omega_{j}^{\prime }\geq 0,\;\text{for}\;\text{all}\;\text{j} \end{array}$$ (9)

The solution to the above problem is the final weights of the criteria and the value of *ξ*^*L*^. *ξ*^*L*^ (values closer to zero show better consistency) is an indicator of the consistency of the comparisons.

#### 3.2.2 The maximal deviation model

This paper adopts the maximal deviation method to determine the weight coefficients because it can automatically obtain the exact and reliable weights without subjectivity. Suppose *Z* = {*Z*_1_, *Z*_2_, …*Z*_*n*_} is the set of cases for multi-attribute decision-making, where *X* = {*X*_1_, *X*_2_, …*X*_*m*_} represents the set of attributes. Let *ω*″ = (*ω*_1_, *ω*_2_…*ω*_*m*_)^*T*^ be the weight vector. *P = (p*_*ij*_*)*_*n×m*_ represents the normalized decision matrix, where *i* ∈ N, *j* ∈ *M*. Let $d_{j}=\sum_{i=1}^{n}\sum_{k=1}^{n}\omega_{j}^{\prime \prime }\left|p_{ij}-p_{kj}\right|,j\in M$, where *d*_*j*_ expresses the total deviation which is relative to *X*_*j*_. The total deviation among all the different attributes *X*_*j*_ with respect to all the cases can be maximized.

If there is no information about the criteria weight, this paper uses the model to determine the weight *ω*″ = (*ω*_1_, *ω*_2_…*ω*_*m*_)^*T*^ as follows:

$$\left\{\begin{array}{c} \text{max}\;d(\omega^{\prime \prime })=\sum_{j=1}^{m}\sum_{i=1}^{n}\sum_{k=1}^{n}\omega_{j}^{\prime \prime }\left|p_{ij}-p_{kj}\right|\\ \sum_{j=1}^{n}{\left(\omega_{j}^{\prime \prime }\right)}^{2}=1,\omega_{j}^{\prime \prime }\geq 0,j\in M \end{array}\right.$$ (10)

Solving the model, and according to $\sum_{j=1}^{m}\omega_{j}^{\prime \prime }=1$, the following result can be derived:

$$\omega_{j}^{\prime \prime }=\frac{\sum_{i=1}^{n}\sum_{k=1}^{n}\omega_{j}^{\prime \prime }\left|p_{ij}-p_{kj}\right|}{\sum_{j=1}^{m}\sum_{i=1}^{n}\sum_{k=1}^{n}\omega_{j}^{\prime \prime }\left|p_{ij}-p_{kj}\right|},j\in M$$ (11)

If the criteria weight is partly known and *O* is the set of incomplete weight information [40], we can also solve the following model to determine the weight vector *ω*″ = (*ω*_1_, *ω*_2_…*ω*_*m*_)^*T*^.


$$\begin{array}{l} \text{max}\;d(\omega^{\prime \prime })=\sum_{j=1}^{m}\sum_{i=1}^{n}\sum_{k=1}^{n}\omega_{j}^{\prime \prime }\left|p_{ij}-p_{kj}\right|\\ \begin{array}{cc} s.t. & \sum_{j=1}^{n}\omega_{j}^{\prime \prime }=1 \end{array}\\ \begin{array}{cc} & \omega_{j}^{\prime \prime }\geq 0,j\in M \end{array}\\ \begin{array}{cc} & \omega_{known}^{\prime \prime }={\left(\omega_{1},\omega_{2}\ldots \omega_{m}\right)}^{T}\in O_{} \end{array} \end{array}$$ (12)

#### 3.2.3 Combination weight

Based on the above two models, the subjective weight *ω*′ = (*ω*_1_, *ω*_2_…*ω*_*m*_)^*T*^ is obtained using the BWM, and the objective weight *ω*″ = (*ω*_1_, *ω*_2_…*ω*_*m*_)^*T*^ is obtained using the maximal deviation method. Then, the final weight $\omega^{*}={\left(\omega_{1}^{*},\omega_{2}^{*}\ldots \omega_{m}^{*}\right)}^{T}$ is calculated using a linear combination (Eq (13)). The value of the *α* parameter is determined by the decision-maker, which indicates the degree of subjective preference as a number between 0 and 1.


$$\omega_{j}^{*}=\alpha \omega_{j}^{\prime }+(1-\alpha )\omega_{j}^{\prime \prime },\;\text{for}\;\text{all}\;\text{j}$$ (13)

The decision-maker can decide which of the two weights will play a more significant role in determining the final weights. Generally, the subjective weight obtained by experts receives more attention. Therefore, *α* = 0.618 makes more sense, which is the golden ratio.

### 3.3 Hybrid similarity measurement method

Aiming at calculating the hybrid similarity between target case *C*^*o*^ and historical case *Z*_*i*_, all local similarities of attributes calculated as mentioned above are aggregated by using weights based on the K-nearest approach. The hybrid similarity is calculated by the formula as follows:

$$SIM(C^{o},Z_{i})=\sum_{j=1}^{m}\omega_{j}^{*}\cdot sim(c_{oj},x_{ij}),i=1,2,\ldots ,n$$ (14)

Where, the closer *SIM*(*C*^*o*^, *Z*_*i*_) is to 1, the greater the similarity between the historical case *Z*_*i*_ and the target case *C*^*o*^.

In order to improve the rationality of emergency decision-making, it is necessary to set a similarity threshold. Let *λ* represent the similarity threshold. In this paper, the similarity threshold *λ* can be calculated by the formula as follows:

$$\lambda =\tau \;\text{max}\{SIM(C^{o},Z_{i})\},0<\tau \leq 1$$ (15)

where *τ* is determined by the decision-maker. When *SIM*(*C*^*o*^, *Z*_*i*_) ≥ *λ*, historical cases and emergency measures will be extracted. The extracted historical cases will form a similar case set Ω. That is, Ω = {*Z*_*i*_|*i* ∈ N*}, N* = {*i*|*SIM*(*C*^*o*^, *Z*_*i*_) ≥ *λ*, *i* ∈ *N*}, in which N* is a set of subscripts for historical cases with reference significance for the target case.

### 3.4 Extraction of similar historical cases

**Fig 1:**
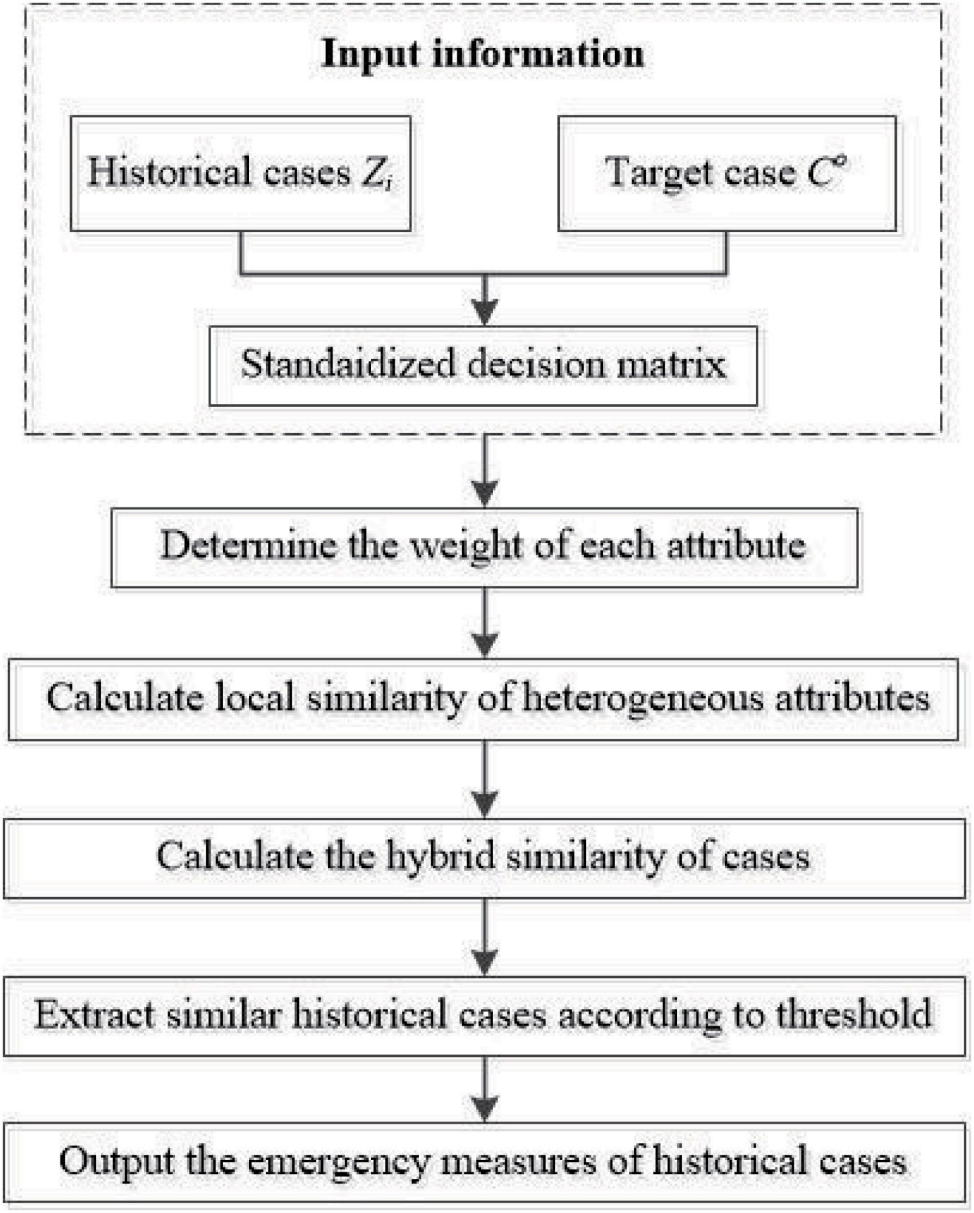
Flowchart of decision-making.

To sum up, this study proposes a CBR-based algorithm based on similarity fusion of multi-source heterogeneous data. The primary extraction process of similar historical cases can be seen in the following flow chart Fig 1.

**Step 1**: Process the data of historical cases and the target case. For hesitant fuzzy linguistic term sets, extend the shorter one by adding the linguistic terms through the method in Definition 2 and get a standardized decision matrix.
**Step 2**: Based on the linear model of BWM in section 3, compute the subjective weight *ω*′ = (*ω*_1_, *ω*_2_…*ω*_*m*_)^*T*^.
With no weight information about the criteria, use Eq (11) to compute the objective weight *ω*″ = (*ω*_1_, *ω*_2_…*ω*_*m*_)^*T*^. With incomplete weight information, use Eq (12) to compute the objective weight *ω*″ = (*ω*_1_, *ω*_2_…*ω*_*m*_)^*T*^.
  Then, the final weights of the decision criteria can be calculated using Eq (13).
**Step 3**: According to Eqs (1)–(4) and (7), use different algorithms to measure the local similarity of heterogeneous attributes involving crisp symbols, crisp numbers, interval numbers, fuzzy linguistic variables, and hesitant fuzzy linguistic term sets.
**Step 4**: Compute hybrid similarity between target case *C*^*o*^ and historical case *Z*_*i*_ with Eq (14), all local similarities of attributes calculated in the third step are aggregated using weights based on the K-nearest approach.
**Step 5**: Depending on the results in the fourth step, let the similarities be arranged in descending order. Extract similar historical cases, which construct a similar case set Ω and output emergency measure of similar cases to provide a reference for the target case.

## 4. Case study

Comprehensive experiments have been conducted to demonstrate the proposed methodology. This paper selects nine urban waterlogging disasters in China, Japan, and Germany as historical cases. It selects the urban waterlogging event which occurred in Zhengzhou, China, on July 20, 2021, as the target case. There are thirteen attributes determined based on the WSR methodology to describe features of urban waterlogging events. The multi-dimensional attributes system of urban waterlogging is shown in Table 1. The Wuli dimension includes the duration of rainfall (*X*_1_), rainfall total (*X*_2_), maximum rainfall per hour (*X*_3_), reservoir operation (*X*_4_), state of the embankment (*X*_5_), and depth of surface accumulated water (*X*_6_). In addition, the Renli dimension includes direct economic losses (*X*_7_), casualties (*X*_8_), range of traffic disruption (*X*_9_), range of communication outages (*X*_10_), and range of power outages (*X*_11_). Finally, the Shili dimension includes the early-warning level (*X*_12_) and relief supplies demand (*X*_13_). These attributes are presented by five data types: crisp symbols, crisp numbers, interval numbers, fuzzy linguistic variables, and hesitant fuzzy linguistic term sets. For the attributes of fuzzy linguistic variables, values are linguistic variables such as very bad(VB), bad(B), medium(M), good(G), and very good(VG). Each of these linguistic variables is represented by a triangular fuzzy number and these numbers are (0, 0, 0.2), (0.1, 0.3, 0.4), (0.3, 0.5, 0.7), (0.6, 0.7, 0.9), (0.8, 1.0, 1.0), respectively. For the attributes described by hesitant fuzzy linguistic term sets, a five-point linguistic scale is constructed, which is S = {s_-2_ = very large, s_-1_ = large, s_0_ = normal, s_1_ = small, s_2_ = very small}. Depending on the data of historical cases (*Z*_1_, *Z*_2_, …, *Z*_9_) and target case(*C*^*o*^), the initial decision matrix is shown in Table 2.

**Table 2:** Initial decision matrix.

	*X*_1_	*X*_2_	*X*_3_	*X*_4_	*X*_5_	*X*_*6*_	*X*_7_	*X*_8_	*X*_9_	*X*_10_	*X*_11_	*X*_12_	*X*_13_
*Z*_1_	3	180	151	M	B	[0.8,2]	13.2	34	{s_-1_, s_0_}	{s_-1_}	{s_-2_}	III	{s_-1_, s_0_}
*Z*_2_	16	215	100.3	B	B	[1,2.2]	116.4	79	{s_-2_, s_0_}	{s_-1_}	{s_-1_, s_0_}	II	{s_-1_}
*Z*_3_	8	100	87.5	B	M	[0.7,1.9]	0.6679	2	{s_0_, s_1_}	{s_0_}	{s_1_, s_2_}	III	{s_0_, s_1_}
*Z*_4_	55	274	75	B	B	[0.7,1.5]	0.8679	5	{s_-1_, s_0_}	{s_-1_, s_0_}	{s_0_, s_1_}	II	{s_-2_, s_0_}
*Z*_5_	72	182.4	23.7	VB	B	[0.5,2]	91	122	{s_-2_}	{s_-2_}	{s_-1_}	I	{s_-2_, s_-1_}
*Z*_6_	72	1852.5	150	VB	VB	[1.0,2.2]	180	180	{s_-2_}	{s_-2_, s_-1_}	{s_-2_}	I	{s_-2_, s_-1_}
*Z*_7_	12	258	125	VB	B	[0.8,1.5]	10	30	{s_-1_, s_0_}	{s_-1_, s_0_}	{s_-1_}	II	{s_-1_, s_0_}
*Z*_8_	24	191.3	98	B	M	[0.5,1.5]	3.64	0	{s_-1_}	{s_-1_, s_0_}	{s_-1_, s_0_}	III	{s_-1_}
*Z*_9_	45	264.4	80	VB	VB	[1.3,1.9]	92	16	{s_0_, s_1_}	{s_-1_, s_0_}	{s_-2_, s_-1_}	III	{s_-1_, s_0_}
*C* ^*o*^	72	640.8	201.9	VB	B	[1.8,2.5]	532	292	{s_-2_, s_-1_}	{s_-1_}	{s_-2_, s_0_}	I	{s_-2_, s_-1_, s_0_}

Based on the reference comparisons in BWM, the priorities of the criteria are determined as numbers between 1 and 9. Table 3 shows the decision-maker preferences.

**Table 3:** Decision-maker preferences.

Criteria	*X*_1_	*X*_2_	*X*_3_	*X*_4_	*X*_5_	*X*_*6*_	*X*_7_	*X*_8_	*X*_9_	*X*_10_	*X*_11_	*X*_12_	*X*_13_
BO (Best criterion: *X*_12_)	3	4	2	5	4	5	6	3	8	9	7	1	8
OW (Worst criterion: *X*_10_)	6	7	8	6	7	5	4	8	2	1	3	9	2

The Decision-making process is as follows.

**Step 1**: Process initial data. For hesitant fuzzy linguistic term sets, extend the shorter one by adding the linguistic terms through the method in Definition 2. The result is shown in Table 4.
**Step 2**: Using the linear model of BWM in section 3 and the pairwise comparison vectors as shown in Table 3, we have: *ω’* = (0.0957, 0.0717, 0.1435, 0.0574, 0.0715, 0.0574, 0.0478, 0.0957, 0.0359, 0.0187, 0.0410, 0.2278, 0.0359)^*T*^, *and ξ*^*L*^ = 0.05 which shows the results consistency.
  Because there is no information about the criteria weight, compute the objective weight of each attribute $\omega_{j}^{\prime \prime }$ based on Eq (11) and the weight vector is *ω”* = (0.1090, 0.0561, 0.0803, 0.0488, 0.0477, 0.0637, 0.0670, 0.0831, 0.0593, 0.0445, 0.0697, 0.2269, 0.0439)^*T*^.
  After calculating the double weights of the criteria based on the BWM method and the maximal deviation, the final weights of the decision criteria can be calculated using Eq (13).
$$\begin{array}{ll} \omega^{*} & =(0.1008,\;0.0657,\;0.1194,\;0.0541,\;0.0624,\;0.0598,\;0.0551,\;0.0909,\;0.0448,\;0.0286,\;0.0520,\\ & 0.2275,\;0.0390{)}^{T} \end{array}$$
**Step 3**: According to Eqs (1)–(4) and (7), compute the local similarity of heterogeneous attributes through different matching algorithms, as shown in Table 5.
**Step 4**: Compute hybrid similarity *SIM*(*C*^*o*^, *Z*_*i*_) between target case *C*^*o*^ and historical case *Z*_*i*_ with Eq (14), and the results are as follows.
$$\begin{array}{ccc} SIM(C^{o},Z_{1})=0.4987 & SIM(C^{o},Z_{2})=0.5193 & SIM(C^{o},Z_{3})=0.4404\\ SIM(C^{o},Z_{4})=0.5107 & SIM(C^{o},Z_{5})=0.7910 & SIM(C^{o},Z_{6})=0.9257\\ SIM(C^{o},Z_{7})=0.5098 & SIM(C^{o},Z_{8})=0.4748 & SIM(C^{o},Z_{9})=0.5212 \end{array}$$
**Step 5**: Depending on the results in Step 4, let the similarities be arranged in descending order.
$$\begin{array}{l} SIM(C^{o},Z_{6})≻SIM(C^{o},Z_{5})≻SIM(C^{o},Z_{9})≻SIM(C^{o},Z_{2})≻SIM(C^{o},Z_{4})≻\\ SIM(C^{o},Z_{7})≻SIM(C^{o},Z_{1})≻SIM(C^{o},Z_{8})≻SIM(C^{o},Z_{3}) \end{array}$$
  Therefore, the sorted result of historical cases is shown below.
$$Z_{6}≻Z_{5}≻Z_{9}≻Z_{2}≻Z_{4}≻Z_{7}≻Z_{1}≻Z_{8}≻Z_{3}$$

**Table 4:** A standardized decision matrix.

	*X*_9_	*X*_10_	*X*_11_	*X*_13_
*Z*_1_	{s_-1_, s_0_}	{s_-1_, s_-1_}	{s_-2_, s_-2_}	{s_-1_, s_-0.5_, s_0_}
*Z*_2_	{s_-2_, s_0_}	{s_-1_, s_-1_}	{s_-1_, s_0_}	{s_-1_, s_-1_, s_-1_}
*Z*_3_	{s_0_, s_1_}	{s_0_, s_0_}	{s_1_, s_2_}	{s_0_, s_0.5_, s_1_}
*Z*_4_	{s_-1_, s_0_}	{s_-1_, s_0_}	{s_0_, s_1_}	{s_-2_, s_-1_, s_0_}
*Z*_5_	{s_-2_, s_-2_}	{s_-2_, s_-2_}	{s_-1_, s_-1_}	{s_-2_, s_-1.5_, s_-1_}
*Z*_6_	{s_-2_, s_-2_}	{s_-2_, s_-1_}	{s_-2_, s_-2_}	{s_-2_, s_-1.5_, s_-1_}
*Z*_7_	{s_-1_, s_0_}	{s_-1_, s_0_}	{s_-1_, s_-1_}	{s_-1_, s_-0.5_, s_0_}
*Z*_8_	{s_-1_, s_-1_}	{s_-1_, s_0_}	{s_-1_, s_0_}	{s_-1_, s_-1_, s_-1_}
*Z*_9_	{s_0_, s_1_}	{s_-1_, s_0_}	{s_-2_, s_-1_}	{s_-1_, s_-0.5_, s_0_}
*C* ^*o*^	{s_-2_, s_-1_}	{s_-1_, s_-1_}	{s_-2_, s_0_}	{s_-2_, s_-1_, s_0_}

**Table 5:** Local similarities of heterogeneous attributes.

	*X*_1_	*X*_2_	*X*_3_	*X*_4_	*X*_5_	*X*_6_	*X*_7_	*X*_8_	*X*_9_	*X*_10_	*X*_11_	*X*_12_	*X*_13_
*Z*_1_	0.3679	0.7688	0.7515	0.5667	1.0000	0.5588	0.3767	0.4133	0.8300	0.8000	0.8000	0	0.8973
*Z*_2_	0.4441	0.7843	0.5654	0.8000	1.0000	0.6765	0.4574	0.4822	0.8626	0.8000	0.8775	0	0.8509
*Z*_3_	0.3955	0.7345	0.5263	0.8000	0.7667	0.5000	0.3679	0.3704	0.6838	0.7879	0.6127	0	0.7559
*Z*_4_	0.7816	0.8112	0.4906	0.8000	1.0000	0.3824	0.3680	0.3742	0.8300	0.8626	0.7354	0	0.9529
*Z*_5_	1.0000	0.7698	0.3679	1.0000	1.0000	0.4706	0.4360	0.5587	0.8775	0.7879	1.0000	1	0.8973
*Z*_6_	1.0000	0.5009	0.7473	1.0000	0.8000	0.6765	0.5156	0.6814	0.8775	0.8626	0.8000	1	0.8973
*Z*_7_	0.4191	0.8038	0.6495	1.0000	1.0000	0.4118	0.3744	0.4077	0.8300	0.8626	0.8000	0	0.8973
*Z*_8_	0.4987	0.7738	0.5582	0.8000	0.7667	0.3235	0.3699	0.3679	0.8775	0.8626	0.8775	0	0.8509
*Z*_9_	0.6762	0.8067	0.5046	1.0000	0.8000	0.6765	0.4369	0.3886	0.6838	0.8626	0.8775	0	0.8973

Suppose *τ* = 0.9. Then, the similarity threshold *λ* calculated using Eq (15) is 0.8331. Consequently, *Z*_6_ meets the threshold condition and constitutes a historical case similarity set Ω = {*Z*_6_}, which means the measures of the historical case with the high similarity can provide proper guidance for the decision-making of the target case. The emergency measures of case *Z*_6_ are shown in Table 6.

**Table 6:** Emergency measures of the similar historical case.

Ω	Emergency measures
Z_6_	y_61_ = ‘The pilots flew over the stricken area and size up the situation.’ y_62_ = ‘More than 3500 firemen and 280 boats were mobilized to rescue the people in the stricken area.’ y_63_ = ‘Stop working, going to school, and halting up the traffic.’ y_64_ = ‘ Government allocated 200 million to support the disaster area.’ y_65_ = ‘Fifty power cars were used to generate power for the victims of urban waterlogging.’

## 5. Comparisons and discussions

In reference [9], only the common combination of three heterogeneous attributes, including crisp numbers, interval numbers and fuzzy numbers, was selected to test the proposed model. In practice, there are far more than these attribute forms. Due to human cognitive limitations, different data sources, and time constraints, a single data type cannot satisfy the needs of the case presentation. The proposed method comprehensively considers the multiple formats of attribute values involved in case retrieval. For a better description of the cases, these attributes are described in five forms in this paper, including crisp symbols, crisp numbers, interval numbers, fuzzy linguistic variables, and hesitant fuzzy linguistic term sets. This paper proposes a novel heterogeneous attribute emergency decision-making model based on CBR, with more forms of expression embedded to further improve the proposed model’s applicability for more complicated emergency decision events.

This paper proposes a novel similarity measurement between hesitant fuzzy linguistic term sets (HFLTSs), considering the difference in the number of linguistic terms between two HFLEs, which is different from the method in [41]. The hybrid similarities SIM(*Co*, *Z*_*i*_) between target case *Co* and historical case *Z*_*i*_ calculated by the method in [36] are shown below.


$$\begin{array}{ccc} SIM(C^{o},Z_{1})=0.5080 & SIM(C^{o},Z_{2})=0.5176 & SIM(C^{o},Z_{3})=0.4256\\ SIM(C^{o},Z_{4})=0.5084 & SIM(C^{o},Z_{5})=0.7871 & SIM(C^{o},Z_{6})=0.8237\\ SIM(C^{o},Z_{7})=0.5073 & SIM(C^{o},Z_{8})=0.4723 & SIM(C^{o},Z_{9})=0.5153 \end{array}$$

The sorted result of historical cases is as follows:

$$Z_{6}≻Z_{5}≻Z_{2}≻Z_{9}≻Z_{4}≻Z_{1}≻Z_{7}≻Z_{8}≻Z_{3}$$

Compared the results computed by two similarity measures for hesitant fuzzy linguistic term sets, the similarity values between cases have changed. The reason is that the hesitance degree of the hesitant fuzzy element has a different influence on the calculation. Although the method in [41] considers the number of membership degrees in HFLEs, defining the hesitance degree based on membership degrees is inadequate. In order to overcome this problem, this study proposes a new hesitance degree considering the ratio of the number between the intersection and the union in HFLEs, which can provide better results. Compared to the method in [41], the model proposed in this paper efficiently avoids information loss and distortion and reduces errors in measurement.

Additionally, this paper proposes an advanced criteria weighting method, which combines the BWM with the maximal deviation method. The factors’ weights in [9] were determined by the analysis hierarchy process(AHP) method. Compared to AHP, the BWM is a method based on the vector that needs fewer comparisons. The BWM method only requires 2*n*-3 comparisons, while AHP needs *n*(*n* − 1)/2 comparisons. In order to avoid the uncertainty of subjective assigning of weights from the experts and extract the accurate information from the available data, this paper uses the maximal deviation method to obtain the objective weight. The combination weight comprehensively considers the prior knowledge of the experts and objective influence factors of indicators.

## 6. Conclusions

Learning from previous emergency measures is an effective approach to deal with the present emergency. According to the characteristics of urban waterlogging emergencies, this paper establishes the multi-dimensional heterogeneous attributes system based on WSR, which can better describe the cases and lay a foundation for the emergency decision-making model. Different similarity algorithms are designed for five data types: crisp symbols, crisp numbers, interval numbers, fuzzy linguistic variables, and hesitant fuzzy linguistic term sets. A hybrid similarity measure between the historical and the target cases is developed to rank and select similar historical cases. It greatly improves the efficiency of the emergency model. The measures of the historical case with the high similarity can provide proper guidance for the decision-making of the target case. Finally, this study empirically tests the proposed model with an example of an urban waterlogging emergency in Zhengzhou, which proves the method is reasonable and scientific. However, there is still room for improvement. This paper only concerns five forms of data. In practice, there are far more than these attribute forms. Moreover, this study assumed that decision-makers have the same understanding of the linguistic terms used. In decision-making problems, decision-makers may use unbalanced linguistic term sets and personalized individual semantics to provide linguistic assessments [42, 43]. Thus, it is necessary to further consider this point in linguistic decision-making. Furthermore, the increase in historical cases will result in the deterioration of the CBR resolution time. Effective management of the case base is necessary, which may be solved by artificial intelligence. In the future, this will be implemented through efforts.
